# Effects of 6-Hydroxyflavone on Osteoblast Differentiation in MC3T3-E1 Cells

**DOI:** 10.1155/2014/924560

**Published:** 2014-03-26

**Authors:** Chien-Hung Lai, Yu-Wei Wu, Shauh-Der Yeh, Yu-Hsaing Lin, Yu-Hui Tsai

**Affiliations:** ^1^Department of Physical Medicine and Rehabilitation, School of Medicine, College of Medicine, Taipei Medical University (TMU), No. 250, Wuxing Street, Taipei 11031, Taiwan; ^2^Department of Physical Medicine and Rehabilitation, Taipei Medical University Hospital (TMUH), No. 252, Wuxing Street, Taipei 11031, Taiwan; ^3^International Institute of Nano Biomedicine, TMU, No. 252, Wuxing Street, Taipei 11031, Taiwan; ^4^Graduate Institute of Medical Sciences, College of Medicine, TMU, No. 250, Wuxing Street, Taipei 11031, Taiwan; ^5^Center for Teeth Bank and Dental Stem Cell Technology, TMU, No. 250, Wuxing Street, Taipei 11031, Taiwan; ^6^Department of Urology, School of Medicine, College of Medicine, TMU, No. 250, Wuxing Street, Taipei 11031, Taiwan; ^7^Department of Urology, TMUH, No. 252, Wuxing Street, Taipei 11031, Taiwan

## Abstract

Osteoblast differentiation plays an essential role in bone integrity. Isoflavones and some flavonoids are reported to have osteogenic activity and potentially possess the ability to treat osteoporosis. However, limited information concerning the osteogenic characteristics of hydroxyflavones is available. This study investigates the effects of various hydroxyflavones on osteoblast differentiation in MC3T3-E1 cells. The results showed that 6-hydroxyflavone (6-OH-F) and 7-hydroxyflavone (7-OH-F) stimulated ALP activity. However, baicalein and luteolin inhibited ALP activity and flavone showed no effect. Up to 50 **μ**M of each compound was used for cytotoxic effects study; flavone, 6-OH-F, and 7-OH-F had no cytotoxicity on MC3T3-E1 cells. Moreover, 6-OH-F activated AKT and serine/threonine kinases (also known as protein kinase B or PKB), extracellular signal-regulated kinases (ERK 1/2), and the c-Jun N-terminal kinase (JNK) signaling pathways. On the other hand, 7-OH-F promoted osteoblast differentiation mainly by activating ERK 1/ 2 signaling pathways. Finally, after 5 weeks of 6-OH-F induction, MC3T3-E1 cells showed a significant increase in the calcein staining intensity relative to merely visible mineralization observed in cells cultured in the osteogenic medium only. These results suggested that 6-OH-F could activate AKT, ERK 1/2, and JNK signaling pathways to effectively promote osteoblastic differentiation.

## 1. Introduction

The bone is a dynamic organ that is continuously molded, shaped, and repaired. Bones undergo a life-long remodeling process that involves osteoclast-mediated bone resorption and osteoblast-mediated bone formation [1]. Osteoporosis is a major bone metabolic disease that occurs with aging, especially during the female postmenopausal life. Osteoporosis is caused by bone remodeling imbalance, specifically the increase in bone resorption and decrease in bone formation [2]. These bone metabolic disorders may result in bone fractures, morbidity, and a shortened lifespan [1]. Osteoporosis prevention and bone mass regeneration remain a challenge.

The antiosteoporosis drugs, such as bisphosphonates, have been used to prevent and treat osteoporosis. However, these drugs do little to increase bone mass, and fractures still occur frequently during treatment [3–5]. Estrogen replacement therapy has been the main form of therapy used to alleviate osteoporosis in postmenopausal patients. However, safety concerns regarding long-term estrogen use and the development of other conditions exist, such as breast cancer and cardiovascular disease [6]. Therefore, finding novel antiosteoporotic agents and dietary supplements that stimulate new bone formation and correct the imbalance between osteoclastic bone resorption and osteoblastic bone formation is desirable.

Flavonoids are naturally occurring compounds found in various vascular plants, vegetables, and herbal medicines, which have biological properties such as antioxidant and antimicrobial activities. Moreover, flavonoids have the potential to reduce inflammation, improve cardiovascular health, suppress angiogenesis, and inhibit tumors [7–9]. Flavonoids include flavones, flavonols, flavanols, flavanolols, flavanones, chalcones, and isoflavones [10]. Among these, isoflavones, derived from soy beans, are the most studied flavonoids that have anti-bone- loss properties [11].

One strategy for preventing and treating osteoporosis is to inhibit bone resorption by osteoclasts. Several studies regarding flavonoids and bone loss prevention have focused on inhibiting bone osteoclast activity [12–14]. A feasible and rapid method to screen a series of compounds with potential antiosteoclastic activity by assessing their ability to inhibit the receptor activator of the NF*κ*B ligand- (RANKL-) induced TRAP secretion of osteoclasts was reported by Wu and associates [14]. Another strategy involves stimulating osteoblastic bone formation. Flavonoid aglycones had been shown to promote differentiation and mineralization of osteoblastic UMR 106 cells* in vitro* [15]. Baicalin also had stimulatory effects on the differentiation of osteoblasts [16]. However, little information concerning the osteogenic characteristics of hydroxyflavones is known. Therefore, this study investigates the effects of those hydroxyflavones on osteoblast differentiation, mineralized matrix deposition, and stimulatory signaling in MC3T3-E1 cells.

This study examined the cytotoxicity of designated hydroxyflavones on MC3T3-E1 cells; their ability to induce osteogenesis of MC3T3-E1 cells by measuring the activity of alkaline phosphatase (ALP), an early osteoblast differentiation indicator; and the signaling pathways activated by the selected hydroxyflavones. Finally, the hydroxyflavone with the greatest potential for ALP activation and with the greatest activation capacity on signaling pathways studied was further examined for its calcium deposition accumulative function in the MC3T3-E1 cells.

## 2. Materials and Methods

### 2.1. Materials

**Figure 1:**
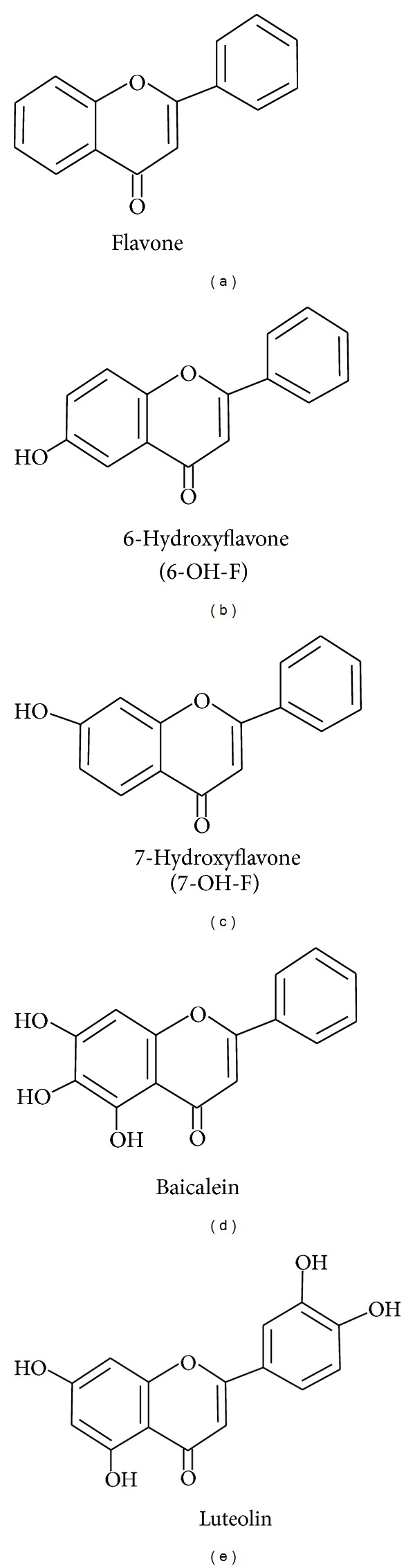
Chemical structure of flavone subset compounds used in this study: (a) flavone, (b) 6-hydroxyflavone (6-OH-F), (c) 7-hydroxyflavone (7-OH-F), (d) baicalein, and (e) luteolin.

The flavones used in this study (Figure 1) were obtained from Sigma-Aldrich (St. Louis, MO, USA). Fetal bovine serum (FBS), anti-anti (Penicillin/Fungizone/Streptomycin), and an *α*-modified minimal essential medium (*α*-MEM) were purchased from Gibco Life Technologies (Grand Island, USA). Trypsin-EDTA, phosphate buffered saline (PBS), sodium tartrate, *β*-glycerophosphate, EDTA, EGTA, triton, sodium pyrophosphate, phenylmethanesulfonyl fluoride (PMSF), Na3VO4, glycine, methanol, ethanol, leupeptin, p-nitrophenyl phosphate disodium salt (pNPP), NaOH, HCl, nature phosphate (NP), fast red (FR), sodium dodecyl sulfate (SDS), dimethyl sulfoxide (DMSO), KCl, NaHCO_3_, NaCl, and 3-(4,5-dimethyl-thiazol-2yl)-2,5-diphenyl tetrazolium bromide (MTT) were all obtained from Sigma-Aldrich (St. Louis, MO, USA). Tris-HCl was purchased from J.T. Baker (Pennsylvania, USA). The antibodies of pERK, pJNK, pAKT, ERK, JNK, and AKT were obtained from Cell Signaling Technology (Danvers, MA, USA).

### 2.2. Cell Culture and Cell Viability Assay

The MC3T3-E1 cells (mouse preosteoblast cell line) were cultivated in the *α*-MEM with a supplement of 10% FBS, 1 x anti-anti, 10 mM *β*-glycerophosphate, 50 *μ*g/mL of ascorbic acid, and 10^-7 ^M dexamethasone at 37°C in a 5% CO_2_ incubator.

The cytotoxic effects of the flavones (flavone, 6-OH-F, 7-OH-F, baicalein, and luteolin) on the MC3T3 cells were evaluated using an MTT assay. The MC3T3-E1 cells were seeded in a 96-well plate at 3000 cells per well and cultured for 48 h. After rinsing with PBS, the cells were treated with various concentrations of the selected flavones in a fresh medium for 24 h. The viable cells were then treated with a newly prepared medium containing 10 *μ*L of 5 mg/mL MTT and 90 *μ*L of the *α*-MEM (10% FBS, 1x anti-anti) in a CO_2_ incubator for 2 h. The MTT was transformed by the living cells to a purple formazan dye which was dissolved in 100 *μ*L DMSO by shaking at 150 rpm for 10 min with an ELISA shaker. Finally, the relative colorimetric intensity of each well was evaluated using a Varioskan flash multimode reader (Thermo Fisher Scientific Inc., MA, USA) at a 570 nm wavelength. The cell viability of the control group without exposure to the flavones was defined as 100%.

### 2.3. Measurement of ALP Activity

The MC3T3-E1 cells were seeded in a 96-well plate, with 10,000 cells per well, and placed in a 5% CO_2_ incubator at 37°C for 4 d. After being rinsed with PBS, the MC3T3-E1 cells were separately treated with 0.25 *μ*M, 1 *μ*M, 4 *μ*M, and 16 *μ*M of the selected flavones (flavone, 6-OH-F, 7-OH-F, baicalein, and luteolin) in a fresh medium for 4 d. After removal of the medium, the cells of each well were fixed with 100 *μ*L of 4% formaldehyde for 10 min. After washing, the cells of each well were treated with 30 *μ*L of pNPP at 37°C for 30 min to determine ALP activity. Afterward, 30 *μ*L of 0.5 N NaOH was added to terminate the reaction. Finally, the conversion of p-nitrophenyl phosphate to p-nitrophenol was determined using an ELISA reader at a wavelength of 405 nm, and the relative intensity of ALP activity over the control group of each well was obtained.

### 2.4. Western Blotting Analysis

After 6-OH-F and 7-OH-F treatment, the whole-cell lysates were prepared with a protein extracting buffer containing 50 mM Tris-HCl (pH 7.4) and 150 mM NaCl (0.1% SDS) and supplemented with protease inhibitors [17]. The proteins were then size-fractionized on SDS-PAGE and transferred to the PVDF membrane. The membrane was blocked with 5% nonfat dry milk prior to incubating with each primary antibody (1 : 1000), which was followed by a respective HRP-conjugated secondary antibody (1 : 2000). After washing with Tris-buffered saline, the membrane was visualized with HRP substrates (Thermo Scientific, Rockford, IL, USA) which were used to label the bound secondary antibody according to manufacturer's protocol. After washing 3 times with Tris-buffer, 1 mL of ECL (Millipore, MA, USA) reagent was added to the membrane for 1 minute in the dark room and the chemiluminescent HRP-labeled specific protein band was visualized by exposing the activated membrane to X-ray film in an X-ray film cassette (Kodak, Rochester, NY, USA). After development, the intensity of the chemiluminescent protein bands was scanned and analyzed by AlphaEaseFCTM 4.0 (Alpha Innotec Corp., San Leandro, USA). The activation of AKT serine/threonine kinases, extracellular signal-regulated kinase (ERK), and c-Jun N-terminal kinase (JNK) was evaluated using primary antibodies specific to the activated AKT, ERK, and JNK, respectively [17].

### 2.5. Calcium Deposition Assay

Calcium deposition of the MC3T3-E1 cells was determined by calcein staining. Briefly, the MC3T3-E1 cells were seeded in a 24-well plate, with 2 × 10^5^ cells per well, and were cultivated in the *α*-MEM supplemented with 10% FBS and 1x anti-anti at 37°C in a 5% CO_2_ incubator for 4 d. The chosen 6-OH-F (40 *μ*M) was then added and cultured for 5 wk with a medium change every 4 d. An aliquot of 0.1 mL 10x calcein-PBS was added to each well without further medium change. The MC3T3-E1 cells were then incubated at 37°C overnight. The cells were fixed with 250 *μ*L of 4% formaldehyde at room temperature (RT) for 15 min. The formaldehyde was then removed, and the cells were washed with PBS followed by staining with 250 *μ*L PBS plus 6 *μ*M DAPI for 5 min at RT. Afterward, DAPI was removed and the cells were rinsed with PBS. Finally, the wells were filled with PBS, and the fluorescent intensity was read with a multiwavelength fluorescent microplate reader. Ex485/Em535 for calcein (green) and Ex340/Em460 for DAPI (blue) were measured, and the calcein data were normalized with DAPI values, respectively. Images of the stained calcium deposition were taken using a fluorescent microscope.

The protocols for determining the total fluorescence absorption of DAPI per well and that of calcein stained, deposited calcium per well, which are at linear dose responses, by the use of a multiple-range fluorescent microplate spectrometer were established recently in our laboratory by Dr. Yu-Wei Wu. This made it possible to normalize the data of total deposited calcium per well by the value of total DAPI intensity per well.

### 2.6. Statistical Analyses

All data were expressed as mean values ± SD (*n* = 6). ANOVA and Duncan's multiple-range test were employed to test significance between groups. A *P* value < 0.05 was considered significant. Values with superscripts of consecutive alphabetical letters are different from each other at *P* < 0.05; the differences are considered statistically significant.

## 3. Results

### 3.1. Cell Viability Assay

**Figure 2:**
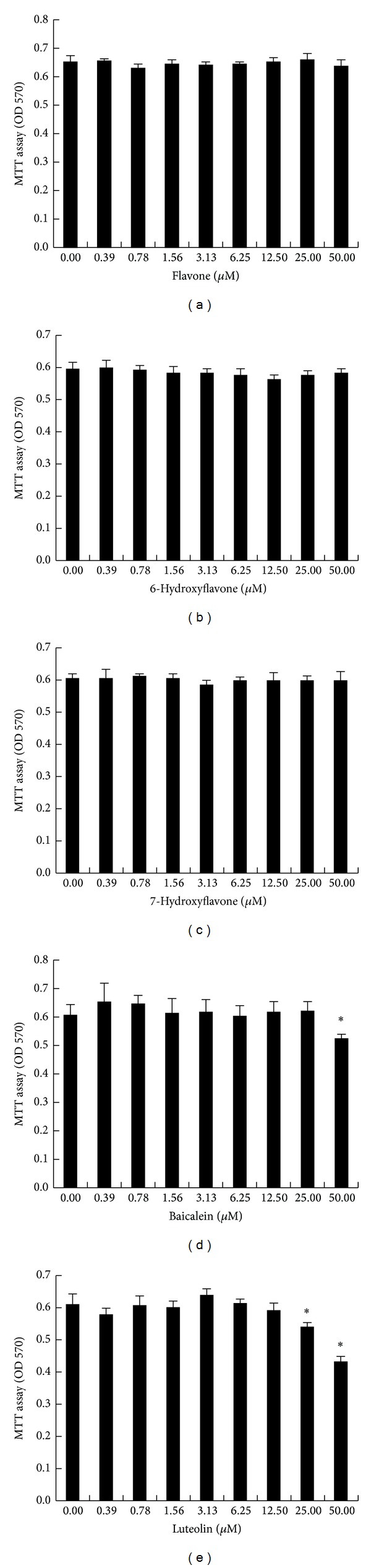
Effects of flavones on MC3T3 cell viability. Effects of the selected hydroxyflavones (flavone, 6-hydroxyflavone (6-OH-F), 7-hydroxyflavone (7-OH-F), baicalein, and luteolin) at various concentrations on MC3T3 cells were examined using an MTT assay. After 24 h of treatment, flavone, 6-OH-F, and 7-OH-F had no obvious effect on the MC3T3 cells. However, 50 *μ*M of baicalein as well as 25 *μ*M and 50 *μ*M of luteolin significantly reduced the number of viable MC3T3-E1 cells compared with untreated cells, indicating cytotoxic effects of these two compounds under the present experimental condition. Data are expressed as mean ± SD (*n* = 6). The statistical significance of each group was analyzed by one-way ANOVA and Duncan's multiple-range test (*P < 0.05, with difference between the current group and untreated group).

An MTT assay was used to determine the cytotoxicity of various concentrations of the selected flavones (flavone, 6-OH-F, 7-OH-F, baicalein, and luteolin) on MC3T3 cells. A dose-dependent decline in cell viability was observed under the present experimental conditions when using 50 *μ*M as well as 25 *μ*M of baicalein and 50 *μ*M of luteolin for 24 h (*P* < 0.05). Other lower doses of baicalein and luteolin treatments did not affect MC3T3 cell viability (Figure 2). Alternatively, the various tested dosages of flavone, 6-OH-F, and 7-OH-F treatments for 24 h did not significantly affect MC3T3 cell viability (Figure 2).

### 3.2. Effect of Hydroxyflavones on ALP Activity in MC3T3-E1 Cells

**Figure 3:**
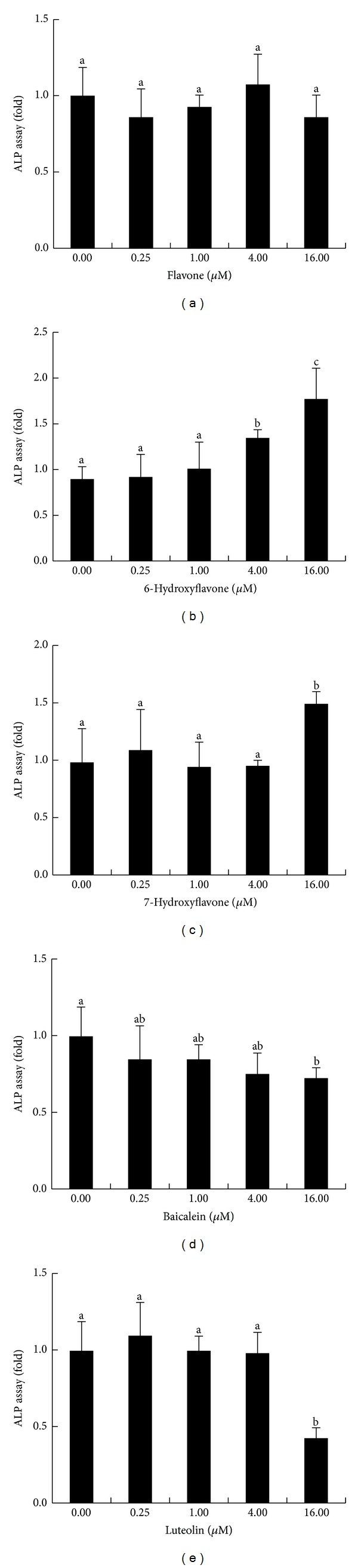
Effects of different hydroxyflavone compounds on the alkaline phosphatase activity (ALP) of MC3T3-E1 cells. Cells were incubated in the presence of various concentrations of flavone (a), 6-hydroxyflavone (6-OH-F) (b), 7-hydroxyflavone (7-OH-F) (c), baicalein (d), and luteolin (e). Data are presented as the mean ± SD (*n* = 6) and are expressed as folds over the control (cells not treated with flavonoids). Columns with consecutive letters (a, b, and c) are statistically different from one another at *P* < 0.05 as determined by ANOVA and Duncan's multiple-range tests.

The cultured MC3T3-E1 cells were treated with different hydroxyflavones at various dosages studied to determine how flavones stimulate and affect the differentiation of MC3T3-E1 cells. ALP activity is an important biochemical marker of differentiated MC3T3-E1 cells, and the effects of hydroxyflavones on ALP activities in MC3T3 cells were first determined. After 4 d of treatment, the cells cultured with flavone showed no significant alteration in ALP activity compared with the control cells (Figure 3(a)). However, cells cultured with 6-OH-F (4 *μ*M and 16 *μ*M) and 7-OH-F (16 *μ*M) showed a significantly higher ALP activity than the control group (Figures 3(b) and 3(c)). However, cells cultured with 16 *μ*M of baicalein or luteolin inhibited ALP activity compared with the control cells (Figures 3(d) and 3(e)).

### 3.3. Hydroxyflavone-Induced Activation of AKT, ERK, and JNK Signaling in MC3T3-E1 Cells

**Figure 4:**
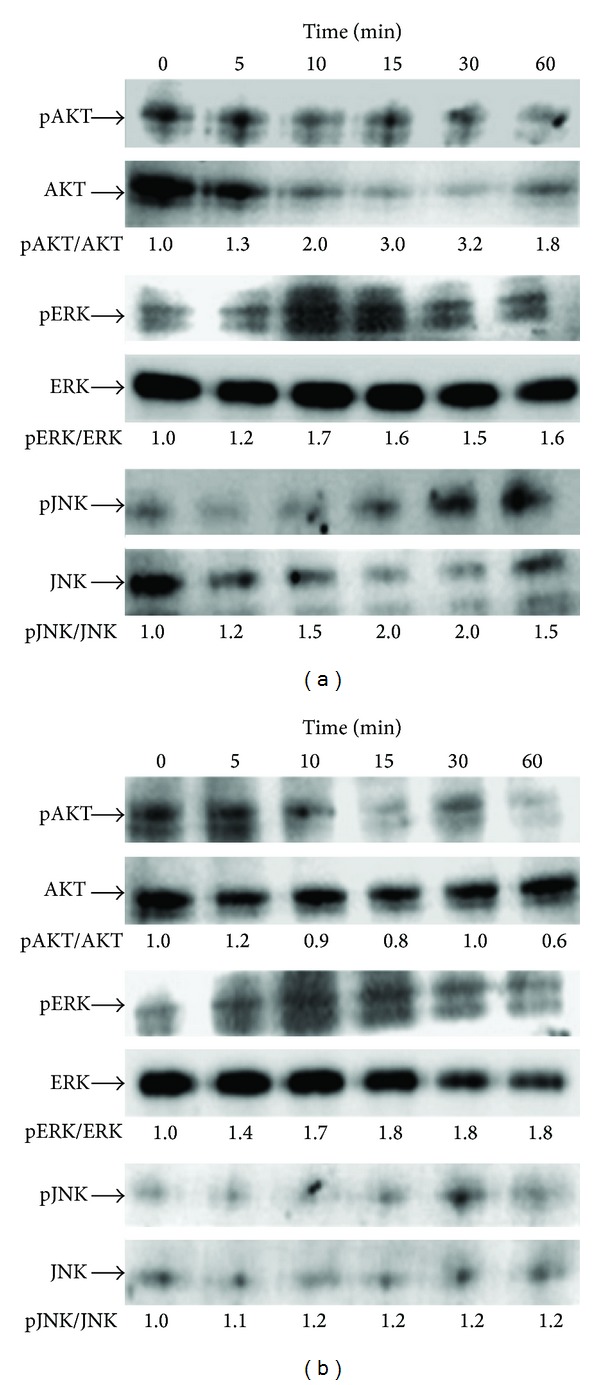
The activation of AKT, ERK, and JNK signaling in MC3T3-E1 cells by 6-hydroxyflavone (6-OH-F) and 7-hydroxyflavone (7-OH-F). Time-related effects of 6-OH-F and 7-OH-F on the phosphorylation of AKT, ERK, and JNK in MC3T3-E1 cells were analyzed using western blotting. The MC3T3-E1 cells treated with 20 *μ*M 6-OH-F or 7-OH-F were collected at designated time points up to 60 min. The patterns of phosphorylated AKT, ERK1/2, and JNK in MC3T3-E1 cells were assessed at 5 min, 10 min, 15 min, 30 min, and 60 min after treatment with 6-OH-F (a) or 7-OH-F (b).

The signaling activated by 6-OH-F and 7-OH-F in MC3T3-E1 cells was determined by analyzing the changes in cellular phosphorylated AKT, ERK, and JNK. The cells were separately treated with 20 *μ*M of 6-OH-F or 7-OH-F and collected at 5 min, 10 min, 15 min, 30 min, and 60 min after treatment. The cell lysates were subjected to western blot analysis. The elevated phosphorylated AKT (~3-fold) and ERK 1/2 (~1.7-fold) were observed at 5 min, 10 min, 15 min, 30 min, and 60 min and at 10 min, 15 min, 30 min, and 60 min after 6-OH-F treatment, with a peak at 15 to 30 min and at 10 min, respectively. Similarly, the increased phosphorylated JNK (~2-fold) was found, in spiting at less detectable intensity, at 10 min, 15 min, 30 min, and 60 min reaching a plateau between 15 and 30 min after 6-OH-F treatment (Figure 4(a)). On the other hand, only weak elevated phosphorylation of AKT (~1.2-fold) caused by 7-OH-F treatment was observed at 5 min and then declined to less than in the control group. A elevation of phosphorylated ERK 1/2 (~1.8-fold) was also detected at 5 min, 10 min, 15 min, 30 min, and 60 min after 7-OH-F treatment but almost no effect on JNK phosphorylation (~1.1- and 1.2-fold) was observed (Figure 4(b)).

### 3.4. Effect of 6-OH-F on Calcium Deposition in MC3T3-E1 Cells

**Figure 5:**
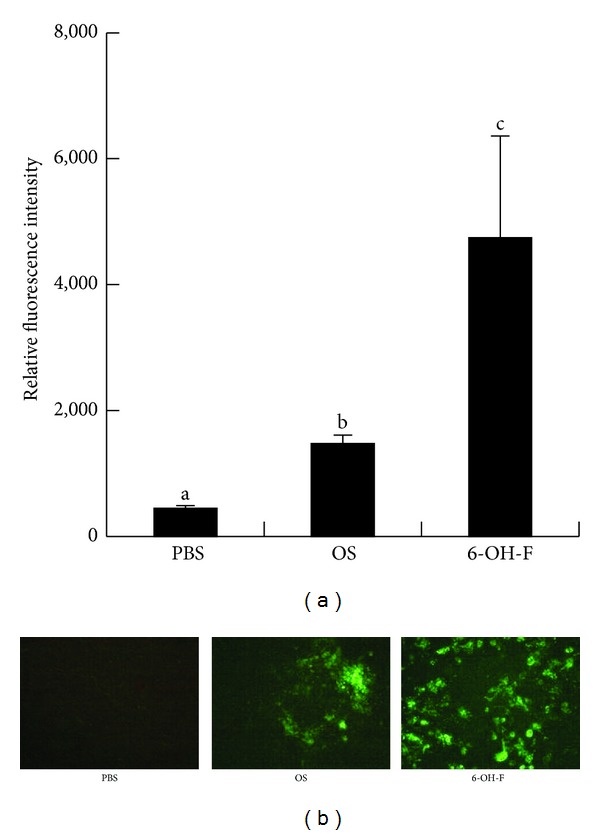
Effects of 6-hydroxyflavone (6-OH-F) on the deposition of the MC3T3-E1 cell mineralized matrix. After 5 wk of treatment with 40 *μ*M 6-OH-F, the mineral matrix deposition was represented as the relative calcein fluorescent intensity per well to the DABI intensity per well: quantification values (a) and images (green fluorescent spots) (b) were summarized. Data are expressed as mean ± SD (*n* = 6). Columns with superscripts of consecutive letters (a, b, and c) are statistically different from one another at *P* < 0.05 determined by ANOVA and Duncan's multiple-range tests, indicating that the difference between the groups with consecutive letters (a, b, and c) is significant. Abbreviation: OS = osteogenic medium; 6-OH-F = 6-hydroxyflavone.

Among the hydroxyflavone subclasses studied, 6-OH-F exhibited the greatest effect on ALP activation and AKT/ERK signaling in MC3T3-E1 cells. Thus, 6-OH-F is chosen to determine its effect on osteoblast differentiation and ability to enhance MC3T3-E1 cell mineralized matrix deposition. MC3T3-E1 cells were examined after being cultured for 5 wk in an osteogenic medium with or without 6-OH-F. The cells were then stained with calcein to identify calcium deposition. There was a significant increase in the green fluorescent intensity of calcein in the 6-OH-F treatment group (*P* < 0.05) relative to merely visible fluorescence in the osteogenic medium control group (Figure 5).

## 4. Discussion

MC3T3-E1 cells (an osteoblast-like cell line could potentially differentiate into osteocytes) were used to investigate the modulatory effects of various hydroxyflavones on osteogenic characteristics These cells provide a useful model for studying bone cell proliferation and differentiation and a renewable culture system to determine the molecular mechanism of osteoblast maturation and the formation of the bone-like extracellular matrix [18].

The activity of ALP is known to be increased in osteoblasts in the early phase of differentiation. ALP is an essential indicator of mineralization capacity, and its expression is a characteristic of osteoblastic cells. ALP activity has been also reported to be a distinguished character of osteogenic cells in culture and to possess the ability to promote bone matrix mineralization [19, 20]. By measuring ALP activity, several hydroxyflavones were first screened for their ability to induce osteogenesis. The results showed that cells cultured with 6-OH-F displayed the strongest stimulatory effect on ALP activity at 4 *μ*M and 16 *μ*M, 1.5-fold and 2-fold enhancement were observed, respectively. Although MC3T3-E1 cells treated with either 6-OH-F or 7-OH-F stimulated a significantly higher ALP activity than control cells in this study, 6-OH-F at both 4 *μ*M and 16 *μ*M showed a dose-dependent increase in ALP activity. However, at a relatively higher dose, 16 *μ*M, 7-OH-F revealed only a 1.5-fold enhancement of ALP activity.

Baicalein and luteolin are also hydroxylated flavones that have been reported to possess anticancer, anti-inflammatory, and antioxidant properties [21–25]. Little information regarding their influence on bone metabolism and osteogenic activity has been found. Kim et al. showed that baicalein stimulated the differentiation of osteoblastic cells [26]. Yang et al. found that luteolin decreased IL-1*β*-induced MMP-9 and -13 mRNA expressions with dose-dependent manner in osteoblastic cell and baicalein had little effect on osteoblastic cells [27]. This study showed that both baicalein and luteolin not only did not stimulate but also suppressed the differentiation of MC3T3-E1 cells. The discrepancy among these studies may be due to the differences in study design, cell type and/or culture system.

Based on the results that 6-OH-F or 7-OH-F increased ALP activity in MC3T3-E1 cells, their effects on osteogenic differentiation were subsequently investigated. The data showed that 6-OH-F stimulated osteoblast differentiation by activating the AKT, ERK 1/2 and JNK signaling pathways. However, 7-OH-F only elevated phosphorylation of ERK 1/2 signaling pathway.

ERK and JNK signaling components, members of the mitogen activated protein kinase (MAPK) superfamily, have been shown to promote osteoblastic cell proliferation and differentiation [28, 29]. This study revealed that 6-OH-F promoted osteoblast differentiation in the present culture condition. These results are in accordance with previous studies that have indicated the involvement of the ERK pathway in stimulating osteoblast differentiation [29–31]. ERK has two isoforms, ERK1 (MAPK3) and ERK2 (MAPK1), both of which are expressed in osteoblasts. ERK phosphorylates RUNX2 to augment its transcriptional activity during early osteoblast differentiation [32]. Xiao et al. found that activation of ERK1/2 by fibroblast growth factor 2 (FGF-2) can increase RUNX2 activation. Interestedly, inhibiting ERK1/2 phosphorylation completely blocked FGF-2 stimulated RUNX2 phosphorylation. FGF-2 stimulated RUNX2-dependent transcription required the ERK pathway [33]. Further, ERK1/2 and MAPK-mediated phosphorylation activated Cbfa1 (also known as RUNX2) phosphorylation in MC3T3-E1 cells [34]. JNK as new signaling pathways involved in BMP-2 induced osteoblastic cell differentiation in the control of ALP expression [35]. Hah et al. showed that JNK signaling played an essential role in the effects of TNF-*α* and IL-1*β* on the ALP expression and mineralization of the periosteal-derived cells. Moreover, ALP expression and mineralization of the periosteal-derived cells were the key factors to determine osteoblast differentiation [36]. The results in this study also demonstrated that 6-OH-F stimulated osteoblast differentiation through the activation of the JNK pathway. This is consistent with other previous reports that JNK pathway activation could promote osteoblast differentiation [35, 37].

AKT and its downstream targets are crucial regulator of osteoblastic differentiation and survival [38, 39]. The resulting data in this study further demonstrated that 6-OH-F treatment elevated phosphorylated AKT level up to more than 300%. Previous study suggested that AKT-mediated signaling pathways could regulate the activity of RUNX2 and promote the gene expression of RUNX2 [39]. Kugimiya et al. have shown that glycogen synthase kinase-3*β*(GSK-3*β*) can negatively regulate RUNX2 activity and impair osteoblast differentiation [40]. And GSK-3*β* phosphorylation is directly inhibited by AKT [41]. Therefore, AKT promoting osteoblast differentiation by stimulating RUNX2 gene expression was suggested by Mukherjee et al. [39].

Because MC3T3-E1 cells cultured with 6-OH-F showed the strongest greatest stimulatory effects on ALP activity, as well as on AKT, ERK, and JNK phosphorylation, 6-OH-F was subsequently used to determine its influence on the deposition of the mineralized matrix by calcein staining. Bone calcein labeling has been used earlier to assess the histomorphometry of remolded bones* in vivo* [42]. Calcein staining has also recently been introduced to examine and quantify osteoblast differentiation and the matrix mineralization of human primary osteoblasts [43]. Further, Calcein, also known as fluorexon or fluorescein complex, is a fluorescent dye with excitation and emission wavelength of 495/515 nm, respectively. Calcein is used for the fluorometric determination of calcium and EDTA titration of calcium in the presence of magnesium. Flavonoids do not exhibit fluorescence at neither 495 nor 515 nm. The results in this study showed a significant increase in calcein staining (~ 3-fold) intensity of mineralization after 5 wk of 6-OH-F induction compared to the much lesser visible staining of the osteogenic medium control group cultured in the absence of 6-OH-F.

MTT assay indicated that cells treated with a dose of 6-OH-F or 7-OH-F below 50 *μ*M exhibited no cytotoxicity. Moreover, 6-OH-F at both 4 *μ*M and 16 *μ*M and 7-OH-F at 16 *μ*M could increase ALP activity. These findings implied that 6-OH-F and 7-OH-F pose no harm under the stimulatory dose to MC3T3-E1 cell differentiation. In addition, the viability of MC3T3-E1 cells in this study declined after 24 h of 50 *μ*M baicalein or 25 *μ*M and 50 *μ*M luteolin treatments. This indicates that baicalein and luteolin may have potential to induce cytotoxicity and lead to cancer cell apoptosis. Baicalein and luteolin have been reported to have a cytotoxic effect on various tumor cells [21, 23]. However, further investigation is required to clarify the optimal baicalein and luteolin doses to inhibit cancer cell formation rather than their cytotoxic effect.

The 6-OH-F compound, a subclass of flavones, is a naturally occurring flavone found in the leaves of* Barleria prionitis*, a plant species in the Acanthaceae family native to India [44]. Antitumor, antioxidant, and anxiolytic-like effects have been found in this compound [44–46]. However, information regarding the osteogenic characteristics of 6-OH-F is not enough or absent. In this study, the osteogenic ability of 6-OH-F was investigated. The results revealed that cells cultured with 6-OH-F significantly increase ALP activity and the calcium deposition of MC3T3-E1 cells via the AKT, ERK, and JNK signaling activation.

The results of this study showed that up to 50 *μ*M of 6-OH-F had no cytotoxicity on MC3T3-E1 cells. Previous study reported that 6-OH-F could bind to type A *γ*-aminobutyric acid (GABA_A_) receptors benzodiazepine (BZ) site with moderate binding affinity [47]. This subtype-selective partial agonist of GABA_A_ receptors exhibited anxiolytic effects without sedative, amnesic, myorelaxant, motor incoordination, or anticonvulsant effects [44]. However, the cytotoxic dose on cortical neuron, other kinds of human cells and* in vivo* still remain unclear and need further investigation.

There are additional limitations to the findings of our study. This study did not extend screening to compare with some easy reaching flavones, such as apigenin and so on. However, previous studies have shown that apigenin can increase osteoblastic differentiation and attenuate osteoclastogenesis and osteoclast function in MC3T3-E1 cells [48, 49]. In addition, although the present study did not directly measure RUNX2 activation, the activation of AKT, ERK, and JNK was found, which has been demonstrated to stimulate RUNX2 expression and/or activation as discussed above, after 6-OH-F treatment. AKT-mediated signaling pathways could regulate the activity of RUNX2 and promote the gene expression of RUNX2 and in turn may promote osteoblast differentiation by stimulating RUNX2 gene expression [39]. ERK could phosphorylate RUNX2 to augment its transcriptional activity during early osteoblast differentiation and ERK phosphorylation could activate RUNX2 phosphorylation in MC3T3-E1 cells [32, 34]. Although no study is included to define the correlation between ERK, JNK, and AKT signaling pathways and RUNX2 expression in present study, the results indeed suggested that 6-OH-F could act via ERK 1/2, JNK, and AKT signaling pathways to increase ALP activity and mineralization of MC3T3-E1 cells.

## 5. Conclusion

Because 6-OH-F facilitates osteoblast differentiation and it is not toxic to MC3T3-E1 cells, this compound may have the potential to reduce or prevent bone loss with fewer side effects. The present findings are the first to illustrate the positive influences that 6-OH-F has on bone metabolism. Although other signaling pathways may be associated with the induction of osteoblastic activity by 6-OH-F, the results suggested that 6-OH-F may stimulate osteoblast differentiation through ERK1/2, JNK, and AKT signaling pathways. Further studies including different dose protocols and* in vivo* research are needed to obtain more information about the clinical treatment capabilities of 6-OH-F.
